# Transcript level of telomerase reverse-transcriptase (*TERT*) gene in the rainbow trout (*Oncorhynchus mykiss*) eggs with different developmental competence for gynogenesis

**DOI:** 10.1007/s13353-024-00887-8

**Published:** 2024-06-26

**Authors:** Konrad Ocalewicz, Marcin Kuciński, Igor Jasielczuk, Artur Gurgul, Mirosław Kucharski, Stefan Dobosz

**Affiliations:** 1 Department of Marine Biology and Biotechnology, Faculty of Oceanography and Geography, University of Gdansk, M. Piłsudskiego 46 Av, 81-378 Gdynia, Poland; 2 Center for Experimental and Innovative Medicine, University of Agriculture in Kraków, Redzina 1C, 30-248, Krakow, Poland; 3 Department of Animal Physiology and Endocrinology, University of Agriculture in Kraków, Mickiewicza 24/28, 30‑059 Krakow, Poland; 4 Department of Salmonid Research, Inland Fisheries Institute in Olsztyn, Rutki, 83-330 Żukowo, Poland

**Keywords:** Aquaculture, Fish reproduction, Gynogenesis, Maternal transcriptome, Telomerase, Telomeres

## Abstract

Expression of the telomerase reverse-transcriptase (*TERT*) gene and activity of telomerase have been reported in the somatic tissues and gonads in fish irrespective of their age and size. Nevertheless, little is known about *TERT* expression in the fish eggs. In the current study, the presence of the *TERT* transcripts was confirmed in the rainbow trout ovulated eggs before and after activation with nonirradiated and UV-irradiated (gynogenesis) sperm. Eggs originating from eight females had high and comparable quality expressed by similar hatching rates. However, survival of the gynogenetic larvae that hatched from eggs activated with UV-irradiated sperm and further exposed to the high hydrostatic pressure (HHP) shock for duplication of the maternal chromosomes varied between females from 2.1 ± 0.4 to 40.5 ± 2.2%. Increased level of *TERT* transcripts was observed in eggs originating from two females, and gametes from only one of them showed improved competence for gynogenesis (27.3 ± 1.9%). In turn, eggs from the female that exhibited the highest survival after gynogenetic activation were characterized by the lowest expression of the *TERT* gene. Telomerase in rainbow trout eggs may compensate erosion of the telomeres during early embryonic development; however, its upregulation does not assure better development after gynogenetic activation.

## Introduction

Telomeres are terminal regions of eukaryotic chromosomes composed of tandemly repeated DNA sequences bound to specific proteins known as shelterins (Blackburn 1991; 2001). Telomeres protect chromosomes from end-to-end fusions and degradation, ensure their complete replication, and enable DNA repair machinery to distinguish between natural chromosomal ends and those resulting from double-strand breaks (DSBs) (Blackburn 2001; de Lange 2002; Chan and Blackburn 2004; Bolzan and Bianchi 2006). On top of that, telomeres could silence the expression of genes located adjacent to the telomeric regions (Pedram et al. 2006). Telomeres shorten after each round of the cell division, and this loss may be compensated by the ribonucleoprotein complex called telomerase. Telomerase is composed of the telomerase reverse-transcriptase subunit (TERT) and the RNA component (TERC) which serves as a template for the telomeric repeats (Greider and Blackburn 1989; Chan and Blackburn 2004). The activity of telomerase is controlled by transcriptional and posttranscriptional regulations of the *TERT* gene expression (Blasco et al. 1996; Bodnar et al. 1998). In addition to the critical role of *TERT* expression/telomerase activity in the telomere elongation process, telomerase is also involved in repair of the chromosomal breaks by synthesis of the telomeric DNA repeats at the doubled-strand breaks (DSBs) (Flint et al. 1994; Nergadze et al. 2007). Several authors have reported that TERT can act as a transcriptional regulator, modulating expression of genes that play crucial roles in many fundamental physiological processes including cell cycle, cell metabolism, cell differentiation, cell signalling, and cell survival (reviewed by Udroiu et al. 2022).

Telomerase activity in humans after birth is exclusively restricted to the germ line cells, stem cells, and tumors (Chan and Blackburn 2004). In turn, expression of *TERT* gene and activity of telomerase have been reported in the somatic tissues and gonads of various model and non-model fish species, irrespective of their age and size (Pfennig et al. 2008; Peterson et al. 2015; Klapper et al. 1998; Elmore et al. 2008; Lund et al. 2009; Anchelin et al. 2011; Henriques et al. 2013; Hartmann et al. 2009). Telomerase in fishes plays important role in the maintaining telomere length during regeneration of the injured tissues (Elmore et al. 2008; Lund et al. 2009; Anchelin et al. 2011). Zebrafish *TERT*^*−*^*/*^*−*^ mutants show severe histopathological abnormalities in their testes, liver, intestines, gills, pancreas, kidney, and muscle tissues what indicates that *TERT* gene in fish is involved in the control of the tissue homeostasis (Henriques et al. 2013).

In zebrafish (Anchelin et al. 2011), *Nothobranchius furzeri* (Hartmann et al. 2009), and platyfish (*Xiphophorus maculatus*) (Downs et al. 2012), ovaries were identified as organs with the highest TERT expression/telomerase activity among examined tissues. Increased expression of the *TERT* gene in the female gonads has been also observed in ovaries of mature diploid rainbow trout (*Oncorhynchus mykiss*), while sterile triploid trout females whose ovaries were significantly reduced and contained only few oocytes, and oogonium exhibited significantly reduced level of the TERT transcript (Panasiak et al. 2023). Moreover, TERT*-*deficient fish are characterized by atrophied ovaries, reduced egg production, and premature infertility (Henriques et al. 2013; Harel et al. 2015). Mentioned above observations suggest that in fishes, telomerase plays also some role in the processes related to the ovarian development and egg production and quality however, our knowledge concerning *TERT* expression in the fish eggs is rather scarce (Hatakeyama et al. 2016).

Induced gynogenesis is a chromosome set manipulation that enables production of fish individuals that inherit exclusively maternal genetic information. Only maternal inheritance is accomplished by activation of fish eggs with homologous or heterologous spermatozoa that nuclear DNA is damaged by UV rays or ionizing radiation. Provided gynogenetic haploids are not viable due to the disturbed gene expression and impaired body development described as haploid syndrome. The diploidy of the gynogenetic zygote may be restored by exposition of the gynogenetically activated eggs to either thermal or high hydrostatic pressure (HHP) shock. Physical shock applied to eggs early after activation results in retention of the 2nd polar body (meiotic gynogenesis) or prevents 1st cell cleavage and results in the generation of fully homozygous gynogenetic doubled haploids (DHs) when applied later around the prophase of the 1st mitosis (mitotic gynogenesis) (Pandian and Koteeswaran 1998). DHs have been utilized in various aquaculture and model fish species to study phenotypic consequences of the recessive alleles, during selective breeding programs, and to generate isogenic and clonal lines (Komen and Thorgaard 2007; Franek et al. 2020). The homozygosity of doubled haploids simplifies detailed linkage analyses, enables identification of the chromosome regions associated with quantitative traits loci (QTL), and radically improves the de novo assembly of genomes sequenced using the next-generation sequencing (NGS) approach (Liu et al. 2012). The wide implementation of mitotic gynogenesis in aquaculture is limited by the low survival of DHs. High mortality of DHs may be only partly explained by the expression of recessive traits and alterations in the egg organelles caused by the sublethal temperatures and hydrostatic pressure shock. It has been observed that efficiency of gynogenesis may also depend on the egg origin (Ocalewicz 2023). Preliminary results provided within comparative transcriptomic analysis of the rainbow trout eggs stripped from different females suggested inter-clutch differences in the *TERT* expression may be linked with the varied ability of eggs for the gynogenetic development (Ocalewicz et al. 2020).

To verify this hypothesis, we induced gynogenesis in eggs provided from eight females. Increased survival of gynogenotes was observed in eggs from four females. The expression of *TERT* gene was assessed in eggs from all clutches. Eggs from two females showed elevated expression of *TERT*, but gametes from only one of these females had increased competence for gynogenesis. In turn, eggs from another female that were characterized by the highest survival after activation with UV-irradiated sperm had very low level of the TERT transcripts.

## Material and methods

The study was carried out in strict accordance with the recommendations in the Polish Act of 15 January 2015 on the Protection of Animals Used for Scientific or Educational Purposes, Journal of Laws 2015, item 266. The protocol was approved by the Local Ethical Committee for the Experiments on Animals in Bydgoszcz (resolution no. 27/2022).

### Fish stocks origin and maintenance

Rainbow trout and European grayling gamete donors came from the broodstocks kept in the Department of Salmonid Research, Inland Fisheries Institute in Olsztyn, Rutki, Poland. Eggs from eight 5-year-old rainbow trout females were collected and kept separately in the plastic bowls at 10 °C pending further procedures. Semen from one 3-year-old rainbow trout and three 3-year-old grayling males was collected into the separate plastic containers. The motility of spermatozoa from each male was confirmed microscopically. and then the semen was stored at + 4 °C until further use.

### Induction of mitotic gynogenesis

**Fig. 1:**
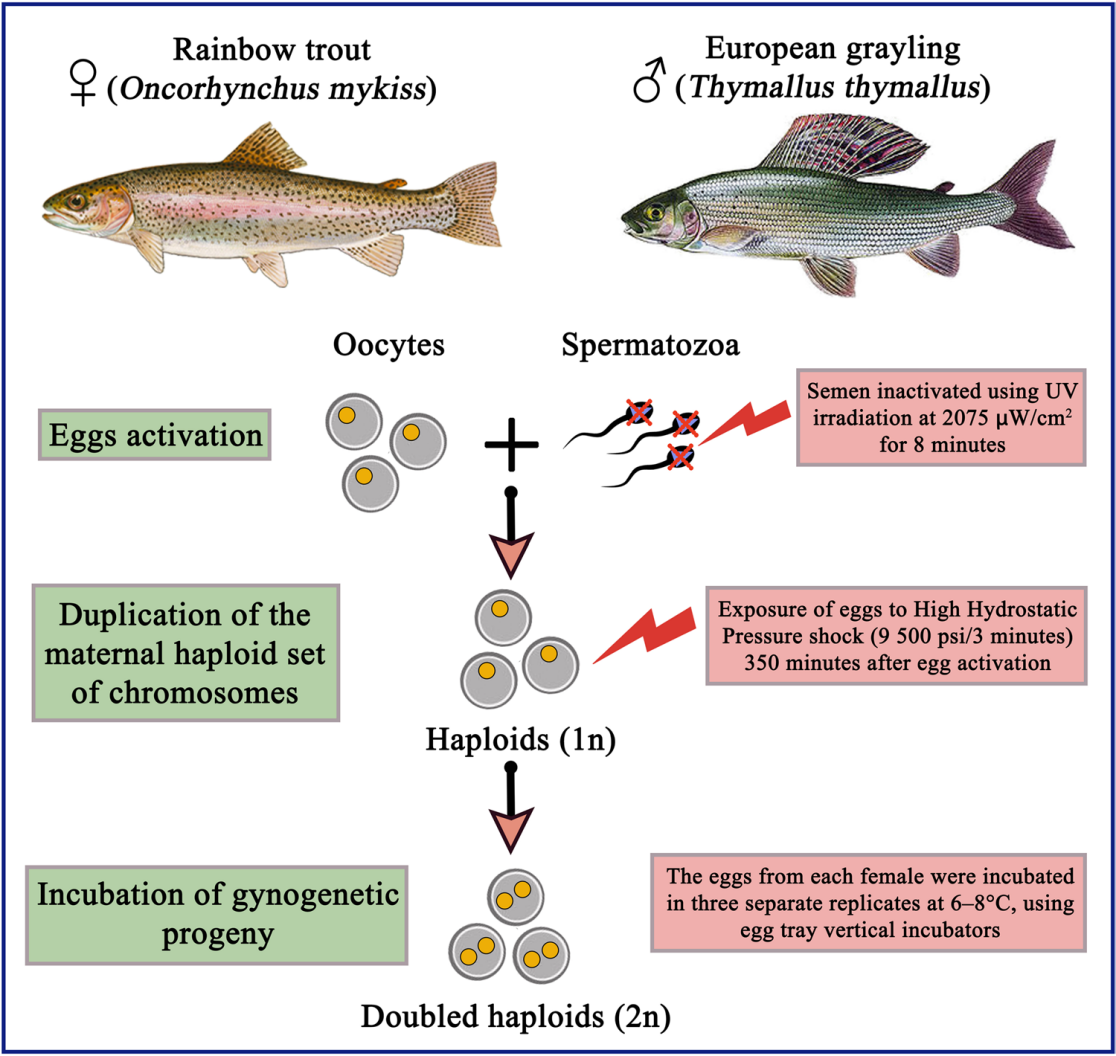
The graphical summary of induced gynogenesis of the rainbow trout (*Oncorhynchus mykiss*) using irradiated European grayling (*Thymallus thymallus*) semen for the egg activation

Mitotic gynogenesis in rainbow trout was performed according to the standard protocol (Jagiełło et al. 2018; Polonis et al. 2018) (Fig. 1). Semen from the three grayling males was mixed and diluted at a ratio of 1:40 in the rainbow trout seminal plasma. A total volume of 15.375 ml of the diluted semen was transferred to a 60-ml glass beaker, placed onto a magnetic stirrer (1400 G) 20 cm under the UV-C lamp (30 W), and irradiated (2075 μW/cm^2^) for 8 min. Batches of eggs (c. 4600) originating from each female were gynogenetically activated with UV-irradiated grayling spermatozoa in the presence of sperm-activating medium (SAM) (154-mM NaCl, 20-mM Tris, 30-mM glycine, 1-mM CaCl_2_, pH 9.0) (Billard 1997). Five minutes after activation, eggs were rinsed with the hatchery water. Eggs activated with the UV-irradiated spermatozoa were exposed to the high hydrostatic pressure (HHP) shock (9500 psi/3 min) exactly 350 min after insemination, using TRC-APV electric/hydraulic device (TRC Hydraulics Inc., Dieppe, Canada). Described above conditions of HHP shock were proven to efficiently restore diploidy in the gynogenetically activated rainbow trout eggs and to generate homozygous doubled haploids (Polonis et al. 2018). Between activation and exposition for the HHP shock, eggs were incubated in a water bath at 10 °C. To establish control groups (C), portions of eggs (c. 350) from each female were fertilized with nonirradiated sperm of the rainbow trout. The eggs from each female and each experimental group were incubated in three separate replicates at 6–8 °C, using egg tray vertical incubators. Dead and live larvae in all groups were counted at hatching, and survival rates were calculated.

### RNA extraction and quantitative real-time PCR

Nonactivated eggs and eggs activated by nonirradiated and UV-irradiated sperm were preserved in RNAlater solution (Sigma Aldrich) within 10 min since stripping and activation, respectively. Following placement in the RNAlater solution, eggs were incubated overnight in a refrigerator (4 °C) and finally stored at − 80 °C until RNA extraction and purification. Approximately, nine eggs from each female were used for RNA isolation, utilizing a modified TRIzol procedure (Ocalewicz et al. 2019). The quality of the obtained RNA was assessed using TapeStation4150 (Agilent Technologies) and the RNA ScreenTape Assay, allowing the evaluation of the RNA integrity number (RIN). High-quality isolates (*RIN* > 8) were quantified using Qubit™ RNA Assay Kits (Thermo Scientific).

The cDNA synthesis was carried out using the High-Capacity cDNA Reverse Transcription Kit (Thermo Fisher Scientific, Waltham, MA, USA) with isolated RNA templates of satisfactory quality. The reaction mixtures were prepared in a total volume of 20 µL, comprising 2.0-µl RT buffer, 0.8 µL of dNTP mix (100 mM), 2.0 µL of RT random hexamer primer (10 ×), 1.0 µL of MultiScribe™ Reverse Transcriptase (50 U/µL), 1.0 µL of RNase inhibitor (50 U/µL), 3.2 µL of nuclease-free, and 200 ng of RNA sample (10-µL volume). The reverse transcription reactions were carried out on a Mastercycler® X50a (Eppendorf, Germany). The samples were incubated for 10 min at 25 °C, followed by 120 min at 37 °C, and the reaction was terminated by heating at 85 °C for 5 min. The resulting cDNA samples were stored at − 20 °C until further qPCR analysis.

Quantitative real-time PCR analysis was conducted using designed primers for the TERT gene (forward: 5′-GCGATCGTAAGCACAGAACA-3′, reverse: 5′-CTCCACTGGCTTCCTGAGAC-3′) and two reference genes, i.e., *ß-actin* gene (*Actb*) (forward: 5′-GCCGGCCGCGACCTCACAAGACTAC-3′, reverse: 5′-CGGCCGTGGTGGTGAAGCTGTAGC-3′) and *elongation factor 1-alpha* gene (*ELF1α*) (forward: 5′-GATCCAGAAGGAGGTCACCA-3′, reverse: 5′-TTACGTTCGACCTTCCATCC-3′). The primer sequences for all genes were designed based on genetic information deposited in the GenBank database (accession numbers: XM_036971724.1, NM_001124235.1, AF498320.1, release 101). All available isoforms and splice variants of the target and housekeeping genes were taken into consideration before primer construction. The primers were designed with the Primer3web (version 4.1.0) with default parameters, including the primer location on the exon-exon junction and a qPCR product length between 130 and 160 bp (Koressaar et al. 2018).

The qPCR was carried out separately for the target gene (*Tert*) and housekeeping genes (*Actb* and *ELF1α*) by a QuantStudio™ 3 Real-Time PCR (Thermo Fisher Scientific™, Waltham, USA) using PowerUp™ SYBR™ Green Master Mix (Applied Biosystems, CA, USA). The real-time reaction mixtures were prepared in a total volume of 10 µL, consisting of 1X SYBR Green Master Mix, 100 nM of each primer, and 1 µL of cDNA. The real-time PCRs were run in triplicates with the following thermal cycling conditions: an initial polymerase activation step at 50 °C for 10 min and then 95 °C for 10 min, followed by 35 cycles of 95 °C for 15 s (denaturation) and 60 °C for 1 min (annealing and elongation). The amplification efficiency for all primer pairs fell within the range of 90–110%. Throughout each run, negative controls involving pure water and non-transcribed RNA were employed to control chemistry contamination and the potential influence of any remaining genomic DNA in the samples. The analysis of the melting curve (60–95 °C) at the end of each run concluded the protocol. Fluorescence data were collected after the elongation step and in 0.1 °C steps on the melting curve. The relative expression was calculated based on the difference between Ct values for reference and target genes using the Livak and Schmittgen’s equation (Livak and Schmittgen 2001). The expression levels (Ct values) of the reference genes were normalized using the geNorm software (Vandesompele et al. 2002).

### Statistical analysis

The differences in survival rates of gynogenotes and fish from the control groups at hatching (26 dpf; days post fertilization) and the fold change expression values of *TERT* relative to the reference genes (*ß-actin* and *elongation factor 1-alpha*) were analyzed using Statistica software v.13.0 (StatSoft Company). Prior to the statistical test selection, data distribution normality and homogeneity of variance were tested by Shapiro–Wilk and Levene’s test, respectively. ANOVA’s HSD Tukey test, or Kruskal–Wallis’ test, was used, based on the data distribution, to determine significant differences in the survival rates and in the telomerase expression between the experimental groups and the individual females within each examined group. Significance was established at the *P* < 0.05 level.

## Results

### Survival rates of hatched larvae

**Fig. 2:**
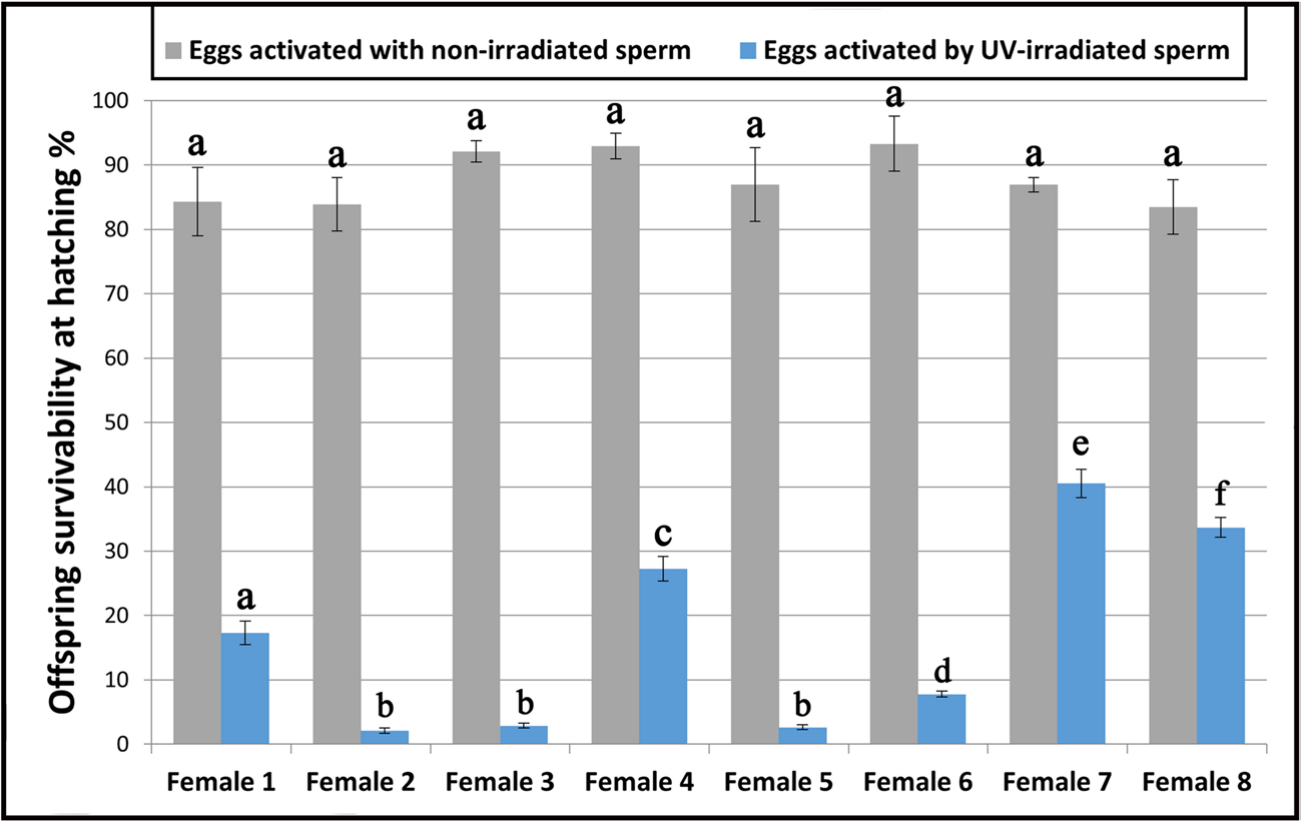
Survival rates of the rainbow trout (*Oncorhynchus mykiss*) offspring that hatched from eggs inseminated with nonirradiated and UV-irradiated sperm. Different letters indicate significant differences (*P* < 0.05) in survival of larvae that hatched from eggs from different females

Eggs from rainbow trout females utilized in the current experiment did not display substantial inter-clutch variations in quality what was demonstrated by the survival rates of hatched larvae from the control groups that ranged from 83.4 ± 4.2 to 93.3 ± 4.3% and did not differ substantially (*P* > 0.05) between females whose eggs were fertilized with nonirradiated milt (Fig. 2). Conversely, the survival rates of larvae that hatched from eggs activated by UV-irradiated sperm and exposed to the HHP shock varied from 2.1 ± 0.4% (female 2) to 40.5 ± 2.2% (female 7), and the differences were significant among most of the females (*P* < 0.05) (Fig. 2). Considering inter-clutch differences in the hatching rates of the gynogenotes, we proposed to divide egg donors into three groups: females whose eggs showed low (< 10%), medium (10–30%), and high (> 30%) survival after gynogenetic activation. Group with low survival of gynogenotes was represented by female 2 (2.1 ± 0.4%), female 3 (2.9 ± 0.4%), female 5 (2.6 ± 0.4%), and female 6 (7.8 ± 0.5%), medium survival: female 1 (17.3 ± 1.8%) and female 4 (27.3 ± 1.9%), and high survival: female 7 (40.5 ± 2.2%) and female 8 (33.7 ± 1.5%). Interestingly, the eggs with the highest survival rate after fertilization with nonirradiated milt (females 3, 4, and 6) did not show any enhanced potential for the gynogenetic development (Fig. 2). In all females, the survival rates of offspring hatched from eggs activated with UV-irradiated semen were markedly lower (*P* < 0.05) when compared to the eggs inseminated with nonirradiated sperm (Fig. 2).

### Transcript level of TERT gene in rainbow trout eggs

**Fig. 3:**
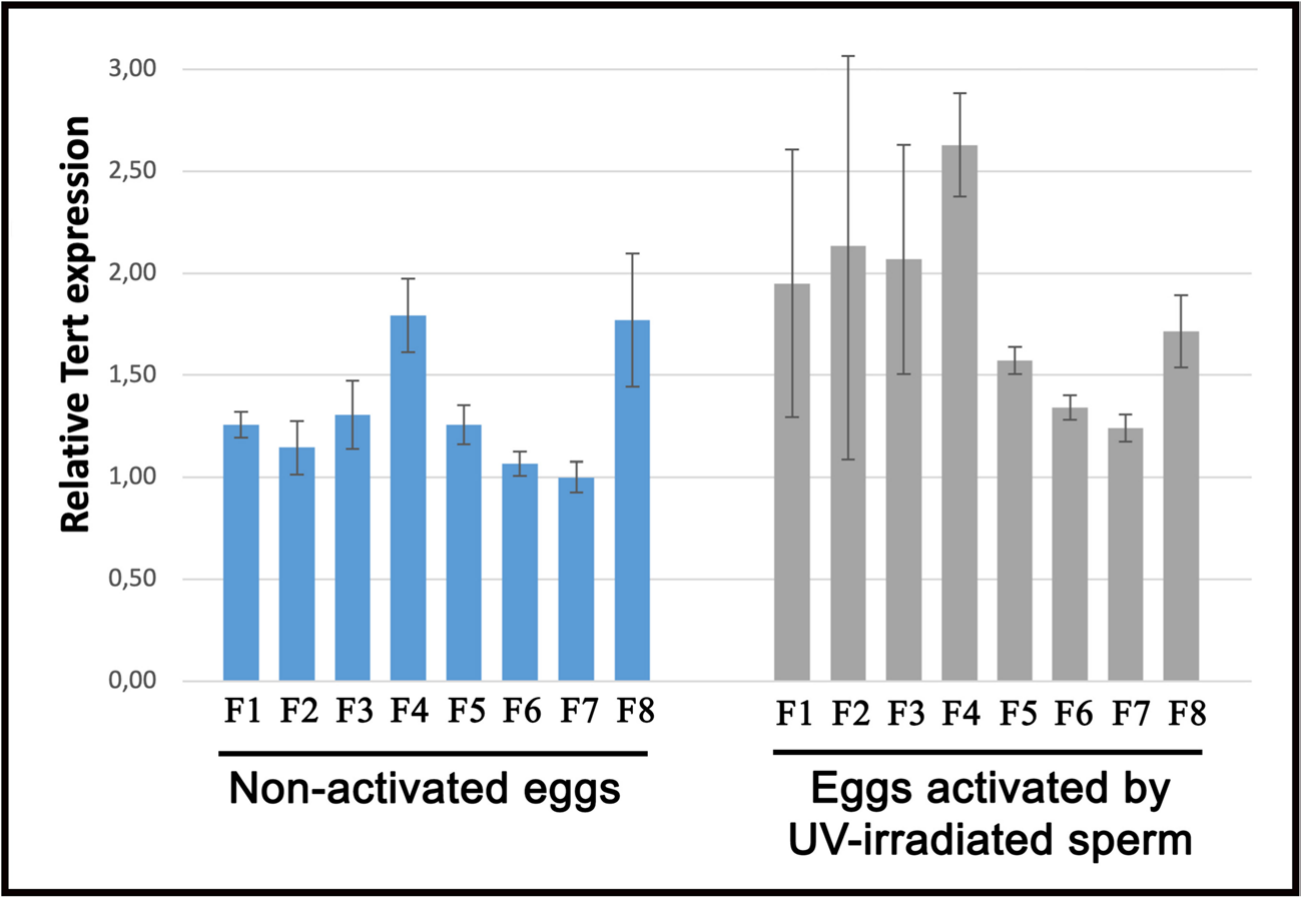
Relative *TERT* mRNA expression in the rainbow trout (*Oncorhynchus mykiss*) eggs. Values are presented as fold changes relative to the reference genes (*ß-actin* and the *elongation factor 1-alpha*). The measure of variation is derived from the respective SEM of the Ct values

Quantitative real-time PCR was used to quantify the TERT mRNAs in the nonactivated eggs and eggs inseminated with nonirradiated sperm (normal development) and UV-irradiated sperm (gynogenetic development). Generally, eggs from most of the clutches displayed similar pattern of the *TERT* expression, and significant alteration in the TERT transcript level was found only in eggs from females 4, 7, and 8 (Fig. 3). Before activation, eggs originated from females 4 and 8 exhibited significantly increased expression of *TERT* gene (*P* < 0.05) when compared to eggs from other females. Inter-clutch differences in *TERT* gene expression were insignificant in eggs that were activated with the nonirradiated sperm. In turn, after activation with UV-irradiated sperm, the only eggs showing significant differences in expression of the TERT gene were those provided from female 4 (elevated expression) and female 7 (decreased expression) (*P* < 0.05) (Fig. 3). Overall, slightly higher (*P* < 0.05) expression level of the TERT gene was detected in eggs activated with nonirradiated and UV-irradiated sperm when compared to nonactivated eggs. No significant (*P* > 0.05) differences in TERT mRNA levels were observed among eggs activated with nonirradiated and UV-irradiated milt.

## Discussion

The current research, for the first time, confirmed the presence of the *TERT* gene transcripts in the rainbow trout ovulated eggs. To date, only a few studies have focused on *TERT* expression and telomerase activity in the female gametes. Initially, telomerase activity in the mature eggs was revealed in *Xenopus* (Mantell and Greider 1994). The activity of telomerase has been also confirmed in oocytes of humans (Wright et al. 1996), rats (Eisenhauer et al. 1997), bovines (Betts and King 1999), mice (Liu et al. 2007), and sea urchin (*Lytechinus variegatus*) (Francis et al. 2006). Alterations in the *TERT* expression and activity of telomerase in oocytes were attempted to link with the gamete quality. In rats and bovines, increased telomerase activity was observed in the small and healthy follicles when compared to the larger and atretic follicles (Eisenhauer et al. 1997; Betts and King 1999). A significant decrease of the *TERT* expression was observed in the mice oocytes during reproductive and postovulatory aging (Yamada-Fukunaga et al. 2013). In addition, telomerase-deficient mice exhibited impaired oogenesis, decreased oocyte quality, increased rate of apoptosis, and impaired chromosome synapsis and aneuploidy (Liu et al. 2002; Liu et al. 2004).

Here, rainbow trout eggs originating from eight females showed similar and high quality, as indicated by the hatching rate that was above 83% (Fig. 2). The expression level of the *TERT* gene was also comparable in these eggs, and only two females (namely nos. 4 and 8) produced gametes with a substantially increased levels of TERT transcripts (Fig. 3). Telomeres of rainbow trout embryos are longer than telomeres in the hatched larvae and juveniles (Panasiak et al. 2020); thus, it might be assumed that high telomerase activity in the rainbow trout eggs ensures the proper length of telomeres and inhibits their erosion during intense cell cleavages in the developing embryos. As telomeres are crucial for the maintenance of the genomic integrity, chromosome segregation, and meiotic recombination, their attrition could decline quality of eggs and reduce developmental competence. Moreover, *TERT* gene and the telomerase activity in the rainbow trout eggs may be part of the mechanism of the double-strand breaks (DSBs) repair. DNA DSBs are the most lethal damages that result in the cell death if unrepaired. DSBs appear after exposure for ionizing radiation (IR), among others. The damaging characteristics of IR make it useful for inactivation of nuclear DNA in the fish eggs when induced androgenetic development (Ocalewicz et al. 2004). Examination of androgenetic rainbow trout and brook trout (*Salvelinus fontinalis*) showed that incomplete inactivation of the egg nuclear genome by ionizing radiation results in the formation of the radiation-induced fragments of the maternal chromosomes that may retain in the cells of the androgenetic individuals. Detailed analysis of such fragments showed that at least some of them have linear structures with DSB ends capped with de novo synthesized telomeric DNAs (Ocalewicz et al. 2004; 2012).

Variation in the survival of gynogenotes developing in eggs originated from different females described in several fish species (Naruse et al. 1985; Komen et al. 1991; Arai 2001; Quillet et al. 1991; Ocalewicz et al. 2020) suggests inter-clutch differences in the egg competence for induced gynogenesis. Rainbow trout eggs used in the present research developed equally after fertilization with nonirradiated spermatozoa, whereas significant inter-clutch differences were observed when eggs were activated by UV-irradiated sperm and exposed to the HHP shock. Exposure of gynogenetically activated eggs to the physical shock around the prophase of the 1st mitosis enables duplication of the maternal set of chromosomes and production of viable DHs that are fully homozygous as both sets of their genomes are identical (Komen and Thorgaard 2007). In the case of rainbow trout eggs, a 3–5-min HHP shock (9000–11,000 psi) applied 350 min after insemination has been proved to efficiently inhibit first cell cleavage leading to duplication of the maternal chromosomes what assures homozygosity of the gynogenetic offspring (Jagiełło et al. 2018; Polonis et al. 2018). Gynogenetically activated trout eggs when not subjected to HHP shock develop as gynogenetic haploids (Hs) and die before hatching (Polonis et al. 2018). The yields of gynogenetic DHs are substantially lower than individuals that hatched from eggs fertilized with nonirradiated spermatozoa (Fig. 2). Large mortality of the gynogenetic DHs results mostly from expression of the lethal alleles and use of eggs with reduced quality (Komen and Thorgaard 2007). Unfortunately, physical treatment that recovers diploidy (and viability) in the gynogenotes may also reduce efficiency of gynogenesis (Michalik et al. 2015) as thermal and HHP shocks not only affect microtubules of the mitotic spindle, but also other cellular organelles and mechanisms connected to the early embryonic development. In the goldfish and crucian carp (*Carassius auratus* Linnaeus 1758), the application of the high-pressure shock and the heat shock to the fertilized eggs resulted in suppression of the dorsoventral differentiation, among other effects. Dorsal deficiencies in the shocked eggs varied between females what suggests that eggs from different females have different sensitivity to the physical shocks (Yamaha et al. 2002). In our study, we tested if eggs with increased competence for gynogenesis have also increased expression of the *TERT* gene. The elevated expression of the *TERT* gene was observed in eggs from females 4 and 8 and so those that had medium and increased survival after gynogenetic activation. However, eggs from female 7 that showed the highest hatching rate after gynogenetic activation were characterized by the lowest TERT expression (Fig. 3). Lack of clear correlation between transcript level of the *TERT* gene in the rainbow trout eggs and efficiency of gynogenesis forces us to revise the original hypothesis about the role of TERT gene expression in the trout eggs with increased competence for the gynogenetic development.

In bovines, oocytes, zygotes, and morula-stage embryos originating from the parthenogenetic activation had lower telomerase activity than blastocysts (Xu and Yang 2001). Here, eggs from some females exhibited a subtle increased level of the TERT transcripts after egg activation. This may be a technical issue as almost the entire maternal mRNA in fish eggs is produced during oogenesis and the early fish embryos are transcriptionally silent until zygotic genome activation (ZGA) that takes place about 10th cell cleavage (Kane and Kimmel 1993). On the other hand, maternal polyA lengths after egg fertilization are dynamic and may change the perceived gene expression level (Liu et al. 2023).

## Conclusions

The presence of transcripts of the telomerase reverse-transcriptase (*TERT*) gene has been confirmed in the rainbow trout ovulated eggs before and after activation with nonirradiated and UV-irradiated (gynogenesis) sperm. Examined eggs that originated from several females had comparable quality; however, survival of gynogenetic embryos developing in eggs from different females differed significantly. Eggs from two females exhibited substantially increased *TERT* transcription; nevertheless, only eggs from one of these females showed improved competence for gynogenesis. In turn, eggs from another female that showed the highest hatching rate after gynogenetic activation were characterized by the lowest TERT expression. Lack of the straight link between level of the *TERT* transcripts in the rainbow trout eggs and efficiency of gynogenesis forced us to reject the hypothesis that trout eggs with up-regulated expression of *TERT* gene show also increased competence for gynogenetic development.

## Data Availability

Data are available upon request.
